# Winter intensity shapes overwintering energy gain and use in bark beetles under range expansion

**DOI:** 10.1242/jeb.251414

**Published:** 2026-01-30

**Authors:** Fouzia Haider, Amanda D. Roe, Mads Kuhlmann Andersen, Yuehong Liu, Antonia E. Musso, Serita Fudlosid, Maya L. Evenden, Heath A. MacMillan

**Affiliations:** ^1^ Department of Biology, Carleton University, Ottawa, ON, Canada, K1S 5B6; ^2^ Natural Resources Canada, Great Lakes Forestry Centre, Sault Ste Marie, ON, Canada, P6A 2E5; ^3^ Department of Biology, Aarhus University, DK-8000 Aarhus, Denmark; ^4^ Department of Biological Sciences, University of Alberta, Edmonton, AB, Canada, T6G 2E9

**Keywords:** Energy reserve, Mountain pine beetle, Overwintering, Cold stress, Climate change

## Abstract

The mountain pine beetle (*Dendroctonus ponderosae*) is an eruptive bark beetle that overwinters as a freeze-avoidant larvae under the bark of pine hosts. In recent years, *D. ponderosae* has undergone a climate change-driven range expansion into previously unsuitable habitats with historically more severe winter conditions. *Dendroctonus ponderosae* overwinters in a non-feeding dormant phase, and energy use is important to post-overwintering fitness. Little is known about how *D. ponderosae* balances energy supply and demand during overwintering. We quantified shifts in energy reserve (supply) and Complex I activity (as an index of demand) in *D. ponderosae* during natural overwintering and simulated early winter onset. We collected *D. ponderosae* larvae from infested lodgepole pine in the autumn (October), winter (January) and spring (April), and sampled a portion of these animals. During autumn and winter, another set of larvae were subjected to either mild overwintering conditions at 6°C or an experimental cold stress of stepwise decreases in temperature to test how an early onset of cold conditions influences the energetic status of overwintering individuals. *Dendroctonus ponderosae* larvae exposed to natural winter conditions accumulated lipids and proteins early in overwintering, which were then available for later use. Early exposure to cold stress in the autumn before full winter acclimatization, however, depleted energy reserves. These findings suggest that the timing and regulation of seasonal acclimatization in *D. ponderosae* have important implications for energy use that can influence subsequent fitness, and thus warming of the overwintering period may facilitate early winter feeding and enhance energy gain of *D. ponderosae* larvae, which could further exacerbate the spread and impact of this pest.

## Teaser

**Highlighted Article:** *Dendroctonus ponderosae* larvae lose energy when early cold hits but gain reserves in warmer winters. As climate change alters seasonal cues, improved overwinter energy balance may boost *D. ponderosae* fitness and accelerate their range expansion.

## INTRODUCTION

The mountain pine beetle (*Dendroctonus ponderosae*; Coleoptera: Curculionidae) is a bark beetle, in western North America, ranging from northern Mexico to northern British Columbia on the west of the Rocky Mountains (Bleiker, 2025; Sambaraju et al., 2012). Winter conditions, especially temperature, play a key role in the biology and distribution of this forest pest (Lester and Irwin, 2012; Stahl et al., 2006). *Dendroctonus ponderosae* undergoes overwintering, a phase in which insects persist through winter in a dormant physiological condition, in a freeze-intolerant state (Delisle et al., 2022; Zachariassen, 1985). After 8–9 months of overwintering in their larval gallery, beneath the bark of their host plants, such as lodgepole and jack pine, the larvae resume development (Bleiker, 2024; Safranyik and Carroll, 2006). Feeding on the phloem tissue of their larval gallery, *D. ponderosae* larvae pupate, and emerge in a synchronized mass flight and attack between late July and mid-August (Safranyik and Carroll, 2006).

Extreme winter temperatures (e.g. below −40°C) contribute significantly to larval mortality and act as a key abiotic constraint on *D. ponderosae* population growth (Bleiker, 2024). During overwintering, larvae use a range of strategies to suppress their supercooling point (Sinclair et al., 2003) and avoid cold injury and mortality (Andersen et al., 2024; Huber and Robert, 2016). *Dendroctonus ponderosae* can also undergo developmental arrest in a larval diapause triggered by decreasing temperatures in the autumn (Bentz et al., 2021; Bentz and Hansen, 2018; Safranyik and Whitney, 1985). Diapause in *D. ponderosae*, however, has been difficult to determine, as they can undergo diapause at different developmental stages including larval, prepupal and adult (Bentz et al., 2021; Bentz and Hansen, 2018; Hansen et al., 2024; Safranyik and Carroll, 2006).

As freeze-avoiding insects, preventing freezing at extreme low temperatures is important for *D. ponderosae* survival and post-winter fitness (Lester and Irwin, 2012), but equally important is the ability to conserve and manage limited energy reserves during this stressful period to support the resumption of development in the spring (Roberts et al., 2023). Insects rely on macromolecular stores of lipids, carbohydrates and proteins in their tissues to survive periods of limited or suspended feeding during winter (Hahn and Denlinger, 2007; Sinclair, 2015). Protecting these energy reserves is crucial, so overwintering insects often reduce metabolic activity in concert with developmental arrest. Nonetheless, basal metabolic processes, stress responses, protein turnover, cryoprotectant synthesis and ion regulation are critical physiological processes that must be maintained throughout winter, which can deplete the finite energy reserves accumulated before winter (Denlinger, 2002; Hahn and Denlinger, 2007; Sinclair, 2015). Therefore, insects minimize energy use during overwintering and, by extension, improve post-winter survival and fitness (Roberts et al., 2023).

Energy metabolism during overwintering relies on the mobilization of energy-rich substrates to sustain essential physiological functions (Roberts et al., 2023). While lipids are the primary energy reserve, carbohydrates and proteins are also critical for processes such as osmoregulation, tissue repair and post-winter metamorphosis (Hahn and Denlinger, 2007; Nestel et al., 2003; Storey and Storey, 2012; Williams et al., 2012). Often during assessment of overwintering energy use, macromolecules other than lipid are overlooked (Noreika et al., 2016). Roberts and Williams (2022) noticed a mismatch while using a dynamic energy use model and validating the model with empirical fat reserves, suggesting the use of other macromolecules such as carbohydrate or protein as an energy source. Storage proteins such as hexamerin are also known to be used as an energy source during food-limited periods in winter (Tian et al., 2021). Together, these macromolecules help combat winter stressors and fuel ATP production (Hahn and Denlinger, 2007; Sokolova, 2021). Therefore, understanding the dynamics of these macromolecules during overwintering and combining this with energy demand is crucial to unveil energy dynamics in overwintering insects.

Over 90% of total energetic demand is provided by mitochondria through the production of cellular ATP via the electron transport system (ETS) (Sokolova, 2018). Additionally, mitochondria play a central role in linking metabolism, stress response and survival (Lubawy et al., 2022). During overwintering when food is assumed to be limited, insects minimize their energy demand and convert stored macromolecules to meet the suppressed energy demands (Hahn and Denlinger, 2007; Roberts et al., 2023). Mitochondrial Complex I, a key entry point of the ETS, plays a central role in meeting these energy demands by controlling the rate of electron flow to synthesize ATP, and is highly sensitive to temperature, which affects thermal tolerance (Iftikar et al., 2014; Jørgensen et al., 2021, 2023). Camus et al. (2017) found that variation in Complex I was strongly related to cold tolerance in flies. Accordingly, Complex I activity is positively associated with overall organismal oxygen consumption (Abele et al., 2007; Thoral et al., 2024), making it a reliable indicator of standard metabolic rate in ectotherms.

Many insects regulate the onset and duration of dormancy in response to both external (e.g. temperature and/or photoperiod) and internal cues (e.g. energy reserves) and adjust their energy acquisition accordingly (Hahn and Denlinger, 2011; Roberts et al., 2023; Short and Hahn, 2023; Williams et al., 2012). Cessation of feeding during winter has largely been studied in animals that have limited or no access to food, such as the spruce budworm (*Choristoneura fumiferana*) or apple maggot (*Rhagoletis pomnella*) (Marshall and Roe, 2021; Ragland et al., 2012). Mountain pine beetles, in contrast, overwinter surrounded by nutrients under the bark of their pine host, yet no study has characterized the temporal energy gain or loss in this species during overwintering. In ectotherms, temperature directly influences energy use as metabolic rates increase with temperature. Warmer winters that increase the metabolic activities of overwintering insects should reduce the energy available for post-winter recovery and development via elevated metabolic rates, while lower winter temperatures would promote energy conservation but raise the risk of cold injury (Irwin and Lee, 2003; Roberts et al., 2023). Climate change disproportionately affects winter condition, including an early winter onset (von Schmalensee et al., 2024). No study, to our knowledge, has been done on the energy balance of overwintering insects in response to early winter onset.

**Fig. 1.**
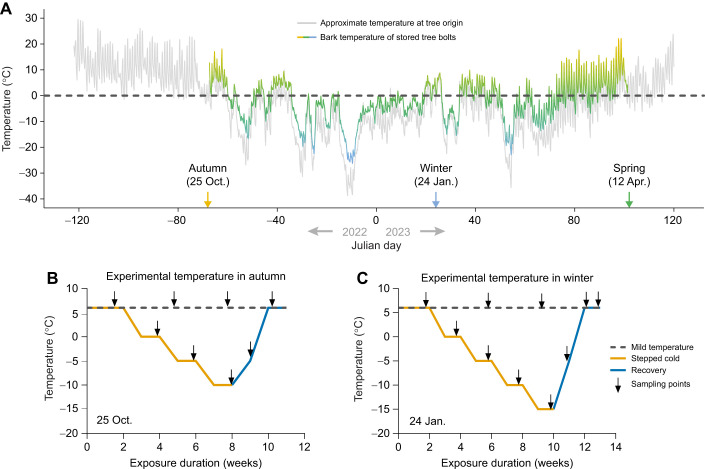
**Experimental design for seasonal and cold-induced experimental treatments.** (A) The autumn (25 October 2022), winter (24 January 2023) and spring (12 April 2023) sampling points, and the approximate temperature at the tree origin (light gray line) and under the bark of the bolts (gradient line) (reproduced from Andersen et al., 2024). (B,C) The winter onset experimental setup for autumn (October 2022) and winter (January 2023), respectively. This experimental setup had 3 groups: (1) mild temperature exposure – mountain pine beetle (*Dendroctonus ponderosae*) larvae were held at a constant 6°C for the entire duration of the experiment (dark gray dashed line), (2) stepped cold stress – larvae were exposed to a −0.3°C min^−1^ ramp and temperature was decreased by 5°C at each step and held for 2 weeks before sample collection (orange solid line) and (3) recovery group – larvae went through a +0.8°C min^−1^ ramp and temperature was held at −5°C for 7 days and then at 6°C for another 7 days before each sample collection (blue solid line). Sample collection points are marked with black arrows.

In this study, we investigated the influence of overwintering conditions on energy dynamics in *D. ponderosae*. We addressed this question using two complementary approaches. First, we assessed changes in macromolecular energy reserves and energy demand across the whole overwintering season (autumn, winter and spring) in field-reared *D. ponderosae* to characterize natural overwintering energetics. We hypothesized that overwintering *D. ponderosae* consume energy stores predominantly in the form of carbohydrates and lipids but preserve protein for post-winter recovery and development. We also hypothesized that *D. ponderosae* suppresses Complex I activity in winter, which would help conserve valuable energy stores. In a second approach, we exposed a subset of *D. ponderosae* to relatively mild temperature conditions at 6°C, beginning in the autumn and winter, and another subset of *D. ponderosae* to progressively lower temperatures over several weeks before recovery to warmer temperatures (Fig. 1). This approach allowed us to test the hypothesis that early onset of winter has negative bioenergetic consequences for *D. ponderosae*.

## MATERIALS AND METHODS

### Insect collection and care

Mountain pine beetle *Dendroctonus ponderosae* (Hopkins 1902) specimens were collected, transported and stored in quarantine before use as described in Andersen et al. (2024). Briefly, lodgepole pine trees (*Pinus contorta*) were baited with synthetic aggregation pheromones (trans-verbenol and exo-brevicomin) and host tree volatiles (myrcene and terpinolene) (#3093, Synergy Semiochemical, Delta, BC, Canada) to attract adult *D. ponderosae* at a field site near Cynthia, AB, Canada (53.34N, −115.48W) over a period from July to August, 2022. Successfully mass-attacked trees were harvested and cut into small sections (40–50 cm long), hereafter called bolts, in October 2022. The cut ends of these bolts were sealed with wax to limit moisture loss and were subsequently transported to the Department of Biological Sciences at the University of Alberta and stored on a sheltered rooftop under ambient natural winter conditions. To monitor the temperature experienced by the beetles, temperature loggers (HOBO 2× External Temperature Data Logger, Onset, Bourne, MA, USA) were inserted approximately 0.5 cm under the bark of the trees. Haphazardly selected bolts were shipped overnight to the Insect Production and Quarantine Laboratory at the Great Lakes Forestry Centre in Sault Ste Marie, ON, Canada, at three different time points: 25 October 2022, 24 January 2023 and 12 April 2023, representing autumn, winter and spring seasons, respectively. At all three time points, bolts were transported (by air, overnight in double-walled secure containers with export authorization from Alberta Agriculture, Forestry and Rural Economic Development, permit #UofA-03-2022) to the PPC-2A quarantine facility. Upon arrival, the bolts were stored at 6°C in total darkness until use in experiments.

### Experimental design

#### Seasonal experiment

Following transport and brief storage at 6°C, *D. ponderosae* larvae were extracted (within the first week of arrival) from larval galleries in two randomly selected bolts from two different trees at each time point (i.e. autumn, winter and spring; Fig. 1A). After larvae collection, the freshly exposed areas of the bolts were sealed with paraffin wax to minimize additional moisture loss and returned to the cold room to be used for the winter onset experiment (see below). All extractions were completed before the bolts warmed to room temperature. We observed mostly late instar larvae, some early instar larvae and a few teneral adults in the bolts we worked with; hence, we conducted our experiment on late instar larvae. The collected larvae were individually weighed, snap-frozen in liquid nitrogen and stored at −80°C. Frozen samples were transported to Carleton University on dry ice for subsequent biochemical analyses.

#### Winter onset experiment

The winter onset experiment was performed using specimens collected from bolts shipped in the autumn (October) and winter (January). Two bolts each from two trees (a total of four bolts) were placed in an ∼100 l chest freezer equipped with a temperature controller (Inkbird ITC-308, 1200 W, Shenzhen, Guangdong, China). The temperature of the freezer was decreased in a stepwise manner from 6°C to 0°C, then to −5°C and lastly to −10°C for the autumn group and with an additional step to −15°C for winter specimens. The temperature was changed at a rate of 0.3°C min^−1^ between each step, with 2 weeks between each step (Fig. 1B,C). The minimum temperatures for autumn (−10°C) and winter (−15°C) were selected to minimize the risk of freezing mortality based on previously measured supercooling points in the same *D. ponderosae* population from the same field site: −15.7±0.4°C (autumn) and −26.7±1.0°C (winter) (Andersen et al., 2024). After being held at the lowest target temperatures for 2 weeks, the temperature of the freezer was increased to −5°C at a rate of 0.8°C min^−1^, held for 7 days, and subsequently returned to 6°C at the same ramp rate, where bolts were held for an additional 7 days (Fig. 1B,C). The relative humidity of the freezer was maintained between 60%  and 70% during the experimental period.

Beetle larvae specimens (6–8 individuals from each bolt) were collected from two bolts at the end of each temperature step using the same extraction and sample preservation methods as in the seasonal experiment. The collected larvae were individually weighed, snap-frozen in liquid nitrogen and stored at −80°C. For the mild temperature condition, the same bolts from seasonal sampling – maintained at 6°C – were used, and larvae were sampled every 2–3 weeks.

### Biochemical analysis of overwintered *D. ponderosae*

We measured water content in a subset of larvae from each treatment group for both seasonal and winter onset experiments. We randomly selected three individuals from every time point and weighed them using a Sartorius ME-5 microbalance scale (accuracy ±1 µg; Precision Weighing Balances, Bradford, MA, USA). After recording the mass, whole specimens in individual weighing dishes were dried in a drying oven at 60°C for 7 days. Larvae mass was recorded on the 4th, 5th and 7th days to confirm a stable mass, indicating that all moisture had evaporated. The water content was estimated as the difference between the fresh and dry mass and expressed as a percentage of body mass. The percentage water content calculated from the subset of measured larvae was used to calculate the dry mass of all the larvae in that treatment group and time point. The dry mass was used to correct the whole-body lipid, carbohydrate and protein content from the two experiments.

Whole-body lipid, carbohydrate and protein (i.e. macromolecules) content was measured for 8–12 individuals in each treatment group and time point. First, larvae were homogenized in ice-cold buffer (0.1 mol l^−1^ Tris-HCl pH 8.0, 0.2% Triton X-100, 153 µmol l^−1^ MgSO_4_ and 0.1 mmol l^−1^ PMSF) in a Fisherbrand™ Bead Mill 24 Homogenizer (Fisher Scientific, Ottawa, ON, Canada). An aliquot of raw homogenate was used for lipid estimation; the rest of the homogenate was centrifuged at 3000 ***g*** at 4°C and the supernatant was used to estimate protein and carbohydrate content. Protein, carbohydrate and lipid were measured in triplicate using colorimetric assay methods modified from Haider et al. (2018) in a microplate reader (Epoch, BioTek, Winooski, VT, USA). Briefly, lipid was extracted using a chloroform–methanol mixture (Folch method) and quantified using the phospho-vanillin method with canola oil as a standard (Van Handel, 1985). Carbohydrate was quantified using the sulfuric acid–phenol method with glucose as standard (Masuko et al., 2005). Total soluble protein was measured using the Bradford assay, according to the manufacturer's instructions (Bio-Rad) with bovine serum albumin (BSA) as a standard (Bio-Rad, Hercules, CA, USA) (Bradford, 1976). Insoluble or methylated proteins are not picked up by the Bradford assay (Brady and Macnaughtan, 2017). Total energy reserve was calculated by transforming the measured protein, lipid and carbohydrate into energy equivalents using their respective energy of combustion: 24 kJ g^−1^ for proteins, 39.5 kJ g^−1^ for lipids and 17.5 kJ g^−1^ for carbohydrates (Gnaiger, 1983).

### Complex I activity determination

The Complex I enzyme activity of the ETS was measured using an aliquot of the supernatant to assess the effect of overwintering on the capacity for energy consumption of *D. ponderosae* larvae. The method was modified from Owens and King (1975) and De Coen and Janssen (1997) to estimate Complex I activity by the rate of 2-(4-iodophenyl)-3-(4- nitrophenyl)-5-phenyl tetrazolium chloride (INT) reduction to formazan at room temperature (23°C) in the presence of NAD(P)H as the electron donor (Haider et al., 2018). Briefly, the samples were diluted with buffer solution (0.13 mol l^−1^ Tris-HCl pH 8.0 and 0.3% Triton X-100) in a 1:8 v:v ratio. In a 96-well plate, 114 µl of the diluted sample and 29 µl of substrate solution [NAD(P)H solution: 1.7 mmol l^−1^ NADH and 250 µmol l^−1^ NADPH] was added. To start the reaction, 57 µl of 8 mmol l^−1^ INT was added to the wells, and the absorbance indicating formazan reduction was measured at 490 nm at 1 min intervals for 15 min or until the reaction reached a plateau at room temperature (varying between 23 and 24°C) in a microplate reader (Epoch, BioTek). To account for potential non-mitochondrial reduction of INT, blanks were run for each sample by replacing NAD(P)H solution with 2 µl of 2 mmol l^−1^ rotenone (mitochondrial Complex I enzyme inhibitor) and 27 µl of 2 mol l^−1^ potassium cyanide (KCN, cytochrome *c* oxidase inhibitor). Complex I activity was blank-corrected and the specific activity of Complex I was calculated based on the extinction coefficient of formazan of 15.9 l mol^–1^ cm^−1^ and corrected for the light path (0.49 cm). The potential energy equivalent was calculated based on the rate of formazan appearance, assuming the stoichiometric equivalent of 1 μmol formazan to 0.5 μmol O_2_, and using oxyenthalpic equivalents for combustion of an average lipid, glycogen and protein mixture (484 J mmol^−1^ O_2_) (Gnaiger, 1983; Haider et al., 2018).

### Statistical analysis

For all statistical tests, assumptions of normality and homogeneity of variance of model residuals were tested using Shapiro–Wilk and Levene's tests, respectively. Statistical outliers (0–1 per season or temperature group), identified through stem-and-leaf plots, were excluded from analyses. If significant main effects for a factor were found, Tukey's HSD *post hoc* test was used for pairwise comparison of the means.

Linear models (LM) were used to assess the effects of treatment on measured bioenergetic parameters. For the seasonal effects on bioenergetic parameters, season (autumn, winter, spring) was used as a fixed factor, and residual dry mass (excluding the macromolecule of interest) was used as a covariate to account for individual variation in body size.

For the effects of stepped cold exposure, time point was used as fixed factor, with residual dry mass included as a covariate. The effect of the tree from which samples were extracted was tested for any random effect and taken out if not significant for the winter dataset. Because of an uneven distribution of beetles collected from each tree or no beetles collected from some individual trees within a time point in the autumn, as well as in the seasonal experiment, ‘tree’ could not be included as a random factor.

Kruskal–Wallis tests were conducted for data that did not meet assumptions of linear models, using season as the grouping factor, to validate the results. Dunn's test was performed for pairwise comparison of the means following the Kruskal–Wallis test.

Unless otherwise noted, all values are reported as means±s.e.m., and a significance level of α=0.05 was used. Statistical analyses were performed using IBM^®^ SPSS^®^ Statistics v.29.0.2.0 (IBM Corp., Armonk, NY, USA) and GraphPad Prism v.10.3.0 (GraphPad Software Inc., La Jolla, CA, USA).

## RESULTS

### Seasonal changes in energy storage and Complex I activity of *D. ponderosae*

**Fig. 2.**
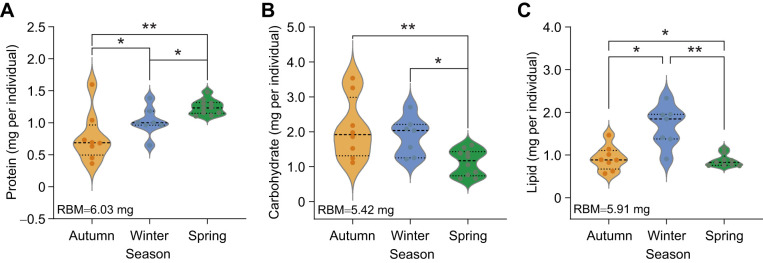
**Effect of seasonality on the stored macromolecules of mountain pine beetle using analysis of covariance.** (A) Protein content, (B) carbohydrate content and (C) lipid content. Autumn samples are presented in orange, winter samples in blue and spring samples in green. Each group contains 7–10 individuals. The data were corrected for residual dry body mass (RBM) for the macromolecule of interest (see Materials and Methods for details), which is shown in the bottom left corner of each panel. Pairwise comparison was done using Tukey's test for protein and carbohydrate, and Dunn's test for lipid data. Statistical significance is indicated by asterisks (*P=0.05 and **P=0.01).

Seasonal temperature shifts resulted in a suite of changes to the stored macromolecules (protein, carbohydrate and lipid) and to the Complex I activity in individual *D. ponderosae* larvae (Table 1). Specifically, the protein content per individual increased (*F*_2,21_=25.3; *P*<0.001) over time from autumn (0.60±0.08 mg) to winter (0.97±0.07 mg) and further into spring (1.42±0.07 mg) (Table 1, Fig. 2A). Conversely, carbohydrate content decreased as autumn progressed into winter and then spring (*F*_2,21_=3.50; *P*=0.049). *Dendroctonus ponderosae* larvae collected in the autumn season had the highest total carbohydrate content (1.96±0.21 mg), followed by larvae collected in the winter (1.83±0.21 mg) and spring (1.25±0.19 mg) (Table 1, Fig. 2B). Seasonality had a pronounced effect on lipid content (*H*_2_=10.29; *P*=0.006; Fig. 2C), with a 130% increase in lipid content from autumn (0.74±0.12 mg) to winter (1.71±0.12 mg), and a 36% decrease as the temperature started to increase in spring (1.09±0.10 mg) (Table 1, Fig. 2C). Lipid content of larvae collected in autumn (*P*=0.02; Dunn's multiple comparison test) and spring (*P*=0.004) was significantly lower than that of winter larvae.

**Table 1.** Linear model results showing the effects of natural overwintering on energy macromolecules and metabolic activity in mountain pine beetle (*Dendroctonus ponderosae*) larvae collected in autumn, winter and spring

Parameter	Seasonal effect				
	Autumn	Winter	Spring	Seasons (main/fixed effect)	Residual dry mass (covariate)
Protein	0.60±0.08	0.97±0.07	1.42±0.07	*F*_2,21_=25.3; *P*<0.001	*F*_1,21_=22.2; *P*<0.001
Carbohydrate	1.96±0.21	1.83±0.21	1.25±0.19	*F*_2,21_=3.50; *P*=0.049	*F*_1,21_=5.20; *P*=0.033
Lipid	0.74±0.12	1.71±0.12	1.09±0.10	*H*_2_=10.29; *P*=0.006	*F*_1,21_=9.23; *P*=0.006
Energy reserves	11.86±0.73	16.60±0.75	14.42±0.52	*H*_2_=13.60; *P*=0.001	*F*_1,21_=3.70; *P*=0.06
Complex I activity	3.72±0.3	1.26±0.12	1.56±0.12	*F*_2,19_=24.5; *P*<0.001	*F*_1,19_=2.37; *P*=0.14

Macromolecules are expressed as mg per individual, energy reserves as kJ g^−1^ residual dry mass and Complex I activity as µmol O_2_ h^−1^ mg^−1^ protein. Residual dry mass was calculated as total dry mass minus the sum of protein, carbohydrate and lipid. Values are presented as means±s.e.m.

**Fig. 3.**
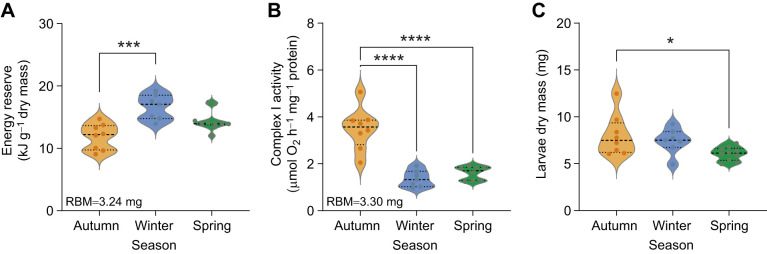
**Effect of natural overwintering on energy reserves, Complex I activity and dry mass of mountain pine beetle using analysis of covariance.** (A) Total energy reserves (estimated by transforming the measured macromolecules to their respective energy equivalent of combustion), (B) Complex I activity (estimated at room temperature) and (C) dry mass (estimated by deducting the percentage water content for the control group of each season). Autumn samples are presented in orange, winter samples in blue and spring samples in green. Each group contains 7–10 individuals. The reserved energy and Complex I activity data were corrected for RBM (indicated in the bottom left corner of each panel). Pairwise comparison was done using Dunn's test for energy reserve, and Tukey's test for Complex I activity and larvae dry mass. Statistical significance is indicated by asterisks (*P=0.05, ***P=0.001 and ****P=0.0001).

By combining the energy equivalent of all three macromolecules measured (protein, lipid and carbohydrate), we found that the total energy budget of *D. ponderosae* larvae changed across seasons (Table 1). The winter-collected samples had approximately 20% more energy reserves (16.60±0.76 kJ g^−1^ dry mass) than larvae collected in any other season (*H*_2_=13.60; *P*=0.001; Table 1, Fig. 3A).

Complex I activity was significantly suppressed from autumn (3.72±0.3 µmol O_2_ h^−1^ mg^−1^ protein) to winter (1.26±0.12 µmol O_2_ h^−1^ mg^−1^ protein) and remained low in spring (1.56±0.12 µmol O_2_ h^−1^ mg^−1^ protein) (*F*_2,19_=24.5; *P*<0.001; Table 1, Fig. 3B).

Seasonality also had a strong impact on water content in the subset of *D. ponderosae* larvae measured (*F*_2,6_=59.15; *P*<0.001; Fig. S1). During autumn and winter, water content was equivalent (54.28±0.5% and 56.09±0.2%, respectively); however, water content in the spring significantly increased to 63.79±0.9% (Fig. S1). The dry mass of larvae decreased throughout overwintering (*F*_2,22_=4.07; *P*=0.03; Fig. 3C); dry mass gradually decreased from autumn (8.02±0.77 mg) to winter (7.41±0.52 mg) to spring (6.06±0.22 mg), such that dry mass was significantly lower in spring compared with the autumn (*F*_2,22_=4.07; *P*=0.03).

### Effect of winter onset on energy storage and Complex I activity of *D. ponderosae* during autumn

**Fig. 4.**
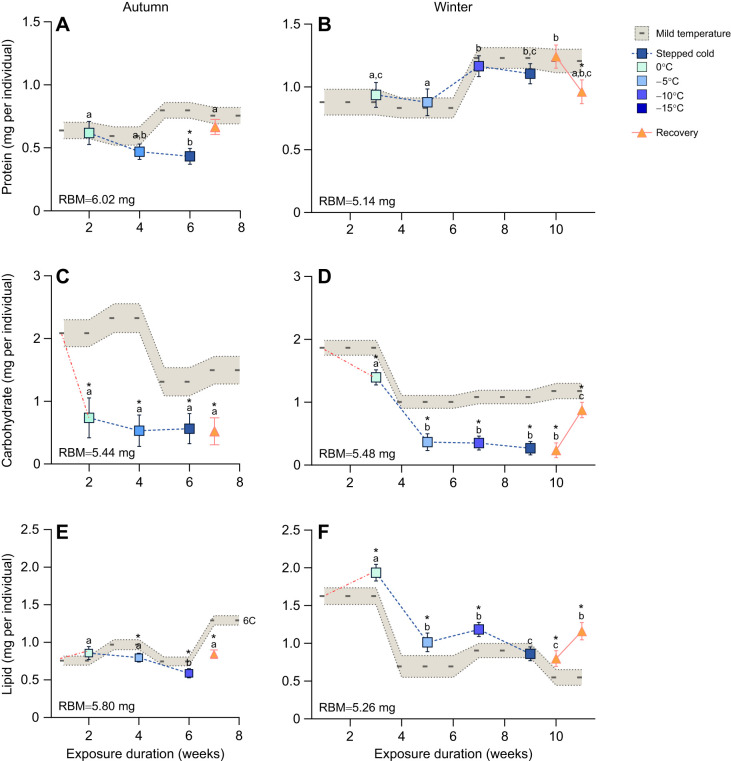
**Effect of mild temperature, stepped cold stress and recovery on the macromolecules of mountain pine beetle larvae in the autumn and winter seasons using a linear model.** (A,C,E) The effect of mild temperature, stepped cold and recovery in the autumn on protein, carbohydrate and lipid content, respectively. (B,D,F) The impact of mild temperature, stepped cold and recovery in the winter on protein, carbohydrate and lipid content, respectively. The dashed line and beige band represent the mean and s.e.m., respectively, of macromolecule levels in larvae maintained at a constant 6°C (mild temperature group). The red dashed–dotted line shows the macromolecule change when the temperature changed from 6°C to 0°C. The blue graduated color indicates the intensity of the cold exposure as the temperature decreased from 0°C to −5°C, −10°C and −15°C. The orange triangles show the recovery group at −5°C for both autumn and winter and 7 days at 6°C in the winter group only. Recovery data for 7 days at 6°C were lost in the autumn group as a result of equipment failure. Each group contains 7–10 individuals. The RBM for each macromolecule of interest was used as a covariate and is indicated in the bottom left corner of each panel. Tukey's *post hoc* test was performed for pairwise comparison. Different letters indicate that values differ significantly (*P*<0.05) within the stepped-cold group at different temperatures. Asterisks indicate that values differ significantly (*P*<0.05) from the mild temperature group.

Exposing the *D. ponderosae* larvae to a constant mild cold (6°C for 8 weeks) in autumn increased stored protein and lipid content per individual, whereas carbohydrate levels decreased over time (Table 2, Fig. 4A,C,E; Table S1). When we simulated an early winter onset with a stepwise temperature decrease, protein and lipid content declined gradually, reaching the lowest levels at −10°C (0.43±0.06 mg and 0.56±0.06 mg, respectively) (*F*_7,53_=3.87; *P*=0.002 and *F*_7,53_=9.15; *P*<0.001, respectively; Table 2, Fig. 4A,E). During the recovery phase of the stepped cold exposure, larvae restored protein content to 0.66±0.05 mg and lipid content to 0.84±0.06 mg in the first 7 days at −5°C (Fig. 4A,E). In contrast to the protein and lipid content, carbohydrate content in *D. ponderosae* larvae declined by 65% as temperature decreased from 6°C to 0°C, then remained low throughout the stepped-cold exposure, and did not increase again during the 7 day recovery at −5°C (*F*_7,53_=10.7; *P*<0.001; Table 2, Fig. 4C).

**Table 2.** Linear model results showing the effects of prolonged overwintering on energy macromolecules (proteins, carbohydrates and lipids) in mountain pine beetle (*D. ponderosae*) larvae across autumn and winter sampling periods

Parameter	Cold-induced stress				
	Autumn (main/fixed effect)	Residual dry mass (covariate)	Winter (main/fixed effect)	Residual dry mass (covariate)	Tree (random factor)*
Protein	*F*_7,53_=3.87; *P*=0.002	*F*_1,53_=34.29; *P*<0.001	*F*_9,74_=3.22; *P*=0.002	*F*_1,74_=20.9; *P*<0.001	n.s.
Carbohydrate	*F*_7,53_=10.7; *P*<0.001	*F*_1,53_=0.22; *P*=0.636	*F*_9,74_=15.3; *P*<0.001	*F*_1,74_=0.32; *P*=0.56	n.s.
Lipid	*F*_7,53_=9.15; *P*<0.001	*F*_1,53_=52.8; *P*<0.001	*F*_9,68_=13.6; *P*<0.001	*F*_1,68_=14.6; *P*<0.001	n.s.
Energy reserves	*F*_7,54_=4.68; *P*<0.001	*F*_1,54_=19.1; *P*<0.001	*F*_9,68_=7.0; *P*<0.001	*F*_1,68_=18.9; *P*<0.001	n.s.
Complex I activity	*F*_7,49_=0.98; *P*=0.45	*F*_1,49_=0.89; *P*=0.34	*F*_11,1.8_=10.8; *P*=0.096	*F*_1,67_=2.13; *P*=0.148	*F*_3,6.9_=6.24; *P*=0.022

Sampling time points were modeled as fixed factors, with residual dry mass as a covariate (autumn and winter) and tree as a random factor (winter only, if significant). Residual dry mass was calculated as total dry mass minus the macromolecules of interest. Values are means±s.e.m. *Tree was not included for autumn because of limited samples.

Based on these changes in protein, carbohydrate and lipid stores, the estimated total energy reserve in *D. ponderosae* larvae experiencing a mild constant cold fluctuated between 11 and 14 kJ g^−1^ dry mass, reaching the highest value of 13.3±0.8 kJ g^−1^ dry mass towards the end of the treatment (Fig. S3A). For larvae that experienced an early onset to winter with the stepwise cold exposure, we observed a decrease in total energy reserves from 11.5±0.7 kJ g^−1^ dry mass at 6°C to 9.0±0.9 kJ g^−1^ dry mass at 0°C; levels remained low throughout the experimental period and showed no increase including during the recovery period at −5°C (*F*_7,54_=4.68; *P*<0.001; Fig. S3A). Despite these changes to total energy reserves, we observed no change in Complex I activity in our constant mild cold and stepwise cold treatments; the activity level stayed between 3.4±0.3 and 4.2±0.3 µmol O_2_ h^−1^ mg^−1^ protein (*F*_7,49_=0.98; *P*=0.45; Fig. S3C).

In the autumn period, body water decreased from 57.8±1.8% to 53±1.2% when larvae were exposed to constant mild cold, but upon exposure to stepped cold treatments, there was a sharp drop of 17% in water content at −10°C, followed by an increase during the recovery at −5°C exposure (*F*_7,15_=10.8; *P*<0.001; Fig. S2A). Dry mass in the constant mild temperature group fluctuated between 7.7±0.33 mg and 6.6±0.35 mg; however, it significantly decreased by approximately 20% upon exposure to 0°C and remained between 5 and 6 mg throughout the stepped cold treatment and recovery period (*F*_7,54_=8.7; *P*<0.001; Fig. S2C).

### Effect of winter onset on energy storage and Complex I activity of *D. ponderosae* during winter

In the winter period, macromolecule content decreased during the constant mild temperature and stepped cold exposure treatments (Fig. 4B,D,F; Table S1). Protein content per individual increased over 11 weeks at the constant mild temperature from 0.8±0.1 mg to 1.2±0.09 mg (Fig. 4B), and a similar change occurred in the protein content of larvae in the stepped cold treatment. We noted a 19% drop in protein content during the recovery phase in the stepped treatment group (*F*_9,74_=3.22; *P*=0.002; Fig. 4B). Stored carbohydrate and lipid content both decreased after 4 weeks at a constant 6°C and remained low for the remainder of this treatment (Fig. 4D,F). In contrast to the constant mild temperature, both carbohydrate (*F*_9,74_=15.3; *P*<0.001; Fig. 4D) and lipid content (*F*_9,68_=13.5; *P*<0.001; Fig. 4F) were significantly affected by the stepped cold exposure. Specifically, carbohydrate content dropped from 1.32±0.1 mg to 0.32±0.1 mg when the temperature decreased from 0°C to −5°C (Fig. 4D). Following this drop, carbohydrate content remained stable at approximately 0.2 mg per individual until after the 7 day recovery at 6°C, when it increased to 0.88±0.1 mg (Fig. 4D). For lipid content, lowering the temperature from 6°C to 0°C resulted in a slight but significant increase in the lipid content per individual (from 1.6±0.1 mg to 1.9±0.1 mg; Fig. 4F). Subsequent temperature decrease caused lipid content to decrease by roughly half (0.82±0.1 mg per individual) at −15°C (Fig. 4F). Similar to carbohydrate content, the 7 day recovery at 6°C led to a partial recovery of lipid content (1.18±0.1 mg per individual; Fig. 4F).

When considered collectively, total energy reserves (i.e. proteins, carbohydrates and lipids) were significantly impacted by time in the constant mild temperature treatment (*F*_9,68_=7.0; *P*≤0.001; Fig. S3B). The initial energy reserves (16.08±0.7 kJ g^−1^ dry mass) dropped to 11 kJ g^−1^ dry mass after 4 weeks at 6°C and remained stable for the remainder of the experiment (Fig. S3B). There was no significant difference between the energy reserves observed in the stepped cold treatment individuals and those in the constant mild treatment. Both groups showed a drop in energy reserves after 4 weeks which stabilized for the remainder of the experiments until the 7 day recovery at 6°C caused a slight increase in reserves (to 13.8±0.7 kJ g^−1^ dry mass; Fig. S3B).

Complex I activity of the *D. ponderosae* larvae exposed at mild temperature remained low (1.2±0.18 µmol O_2_ h^−1^ mg^−1^ protein) at the beginning of the experimental period and increased slightly by the end (2.6±0.2 µmol O_2_ h^−1^ mg^−1^ protein; *F*_11,1.8_=10.8; *P*=0.096; Fig. S3D). Compared with the mild temperature treatment, the stepped cold exposure did not affect Complex I activity, until the final 7 day recovery time point when we observed a sharp increase in enzyme activity from 1.2±0.18 µmol O_2_ h^−1^ mg^−1^ protein to 3±0.18 µmol O_2_ h^−1^ mg^−1^ protein (Fig. S3D).

Exposure to a constant mild temperature in winter caused a gradual increase in water content from 56.56±2.70% to 65.88±2.70%, while dry mass decreased from 8.08±0.20 mg to 6.26±0.20 mg in *D. ponderosae* larvae (Fig. S2B,D). By contrast, water content stayed stable (around 55%) during the stepped cold treatment, and increased during the recovery period at −5°C and 6°C (to 61.8±2.7% and 60.4±3.3%, respectively; Fig. S2B). The dry mass of larvae did not significantly change during the stepped-cold exposure compared with the constant mild temperature (Fig. S2D).

## DISCUSSION

Energy reserves in *D. ponderosae* larvae changed during natural overwintering and dynamically responded to changes in temperature in the laboratory. Under natural winter conditions, the three primary macromolecules (protein, carbohydrate and lipid) and total energy reserves showed substantial seasonal variation, including phases of accumulation. Specifically, we found that *D. ponderosae* larvae accumulated lipids as winter approached, which drove an overall increase in their total energy reserves during a period in which we expected larvae to be dormant and non-feeding. Complex I activity was suppressed in both winter and spring, and larvae had lower body mass and higher water content by spring in concert with a substantial drop in lipid stores. In the winter onset experiment, we noted that autumn and winter larvae exposed to mild temperature (6°C) showed similar patterns of change in protein and carbohydrate content, while changes in lipid content differed between the autumn- and winter-collected specimens throughout the experimental time points. At mild temperature (6°C), lipid content showed an increasing trend during autumn (Fig. 4E) and a decreasing pattern during winter (Fig. 4F). Larvae exposed to stepped cold stress used carbohydrates in both autumn and winter but were less dependent on proteins and lipids. Through all of this, Complex I activity remained unchanged in response to cold, and total energy reserves decreased markedly during the winter time point. Overall, these findings suggest that mild autumn conditions benefit *D. ponderosae* by allowing them to continue accumulating energy reserves prior to overwintering. An early onset of winter, however, may limit this reserve buildup, creating an energy constraint that could compromise survival and fitness.

### Seasonal shifts in energetics reflect complex overwintering strategies of *D. ponderosae*

Seasonal shifts in energy reserves suggest that there is a nuanced and dynamic interplay between body composition, metabolic regulation, overwintering behavior and physiology in *D. ponderosae*. Specifically, *D. ponderosae* larvae accumulate energy stores in the autumn as part of the preparatory phase for overwintering. This preparation includes a buildup of lipid reserves, moderate depletion of carbohydrate reserves and the suppression of Complex I activity, which collectively suggests a metabolic shift as larvae transition towards dormancy and prepare for harsher winter conditions. Larval dry mass remained unchanged in autumn and winter but declined in spring (Fig. 3C), reflecting a strategic maintenance of energy reserves to support survival and post-winter development, similar to processes observed in other insects such as paper wasps, *Polistes* spp*.* (Stabentheiner et al., 2024). Interestingly, our data diverge from measurements of adult *D. ponderosae* across seasons, which show a more consistent reduction in dry mass (Lester and Irwin, 2012), suggesting life stage-specific overwintering strategies in this species.

Reducing body water can help overwintering insects avoid internal ice formation and is an essential step when preparing for dormancy (Zachariassen, 1991). Seasonal dehydration occurs in other freeze-avoidant insect species, such as the emerald ash borer (*Agrilus planipennis*) and the spruce budworm (*Choristoneura fumiferana*) (Crosthwaite et al., 2011; Marshall and Roe, 2021). In stark contrast to these other species, however, water content in *D. ponderosae* larvae increased steadily from autumn to spring (Fig. S1), as similarly observed in overwintering adult *D. ponderosae* (Lester and Irwin, 2012). Despite this, *D. ponderosae* have very low supercooling points during overwintering (Andersen et al., 2024), suggesting that dehydration is not a necessary component for freeze avoidance in this species. This suggests that *D. ponderosae* lowers its supercooling point through other processes, such as cryoprotectant synthesis and compatible osmolyte accumulation, removing ice nucleating agents, or purging their gut contents as temperature starts decreasing.

Our macromolecule data provide further critical insights into the overwintering strategy of *D. ponderosae*. Notably, between October and January, naturally overwintering *D. ponderosae* larvae accumulate both proteins and lipids (Fig. 2A,C), resulting in an overall increase in total energy reserves (Fig. 3A). Accumulation of protein and lipid requires feeding (Bleiker and Six, 2007; Safranyik and Whitney, 1985); thus, we interpret this accumulation as continued feeding during the mild autumn period (ambient temperature 6–15°C; Fig. 1A). Given that *D. ponderosae* acquire nutrients from both tree phloem and symbiotic fungi (Goodsman et al., 2012), this would account for increases in protein and lipid, as the symbiotic fungi provide an important source of both nutrients (Ayres et al., 2000). Goodsman et al. (2012) showed that fertilizing harvested bolts infested with *D. ponderosae* increased the growth rate of the beetles as a result of feeding. If *D. ponderosae* larvae continue feeding well into the autumn when temperatures are cool but permissive, then continued autumn and winter warming may benefit *D. ponderosae* in its native and expanded range by allowing for extended feeding into the late autumn and winter season, thus permitting further accumulation of energy to be used for dormancy or to resume development in the spring. Future studies on the nutrient status of the tree phloem in response to winter temperature would be a promising direction.

Lipid accumulation is a hallmark of overwintering preparation (Enriquez and Visser, 2023), with lipids in the fat body serving as both energy reserves and signaling molecules (Kühnlein, 2012). Insects can synthesize lipids from carbohydrate and protein precursors (Sinclair and Marshall, 2018), which may explain the lack of increase in carbohydrate from autumn to winter in our dataset (Fig. 2B). We speculate that carbohydrates acquired during this period were rapidly converted into cryoprotectants (e.g. glycerol) or used in lipogenesis, and thus were not picked up in our carbohydrate assay. This conversion of carbohydrate to cryoprotectants would be consistent with previous studies in other freeze-avoiding insects (Crosthwaite et al., 2011; Koštál et al., 2014).

By spring, both lipid and carbohydrate reserves were markedly depleted (Fig. 2B,C), likely reflecting the energetic demands of overwintering and the onset of developmental reactivation (Enriquez and Visser, 2023; Safranyik and Carroll, 2006). Despite this depletion, Complex I activity remained suppressed compared with autumn levels (Fig. 3B), indicating that Complex I activity does not immediately rebound with warming temperatures. This is not surprising as overwintering insects can use a range of substrates – including succinate, proline and glycerol – for ATP production via mitochondrial complexes beyond Complex I, such as Complex II (succinate dehydrogenase), proline dehydrogenase and glycerol-3-phosphate dehydrogenase (Menail et al., 2022; Storey and Storey, 2012; Teulier et al., 2016). These alternative pathways may become increasingly important during spring as energy demands rise in preparation for pupation. Therefore, *D. ponderosae* larvae could also be using alternative energy production pathways as the temperature starts warming up before they become fully active and transition to pupae.

Together, these observations suggest that *D. ponderosae* employ a flexible metabolic program that balances energy storage, conservation and substrate switching across the overwintering period. Autumn and early winter appear to be a phase of active resource accumulation and metabolic reorganization, while mid-winter is characterized by resource conservation that appears to be driven more directly by temperature than by any substantial metabolic suppression, although this remains to be tested explicitly. In spring, *D. ponderosae* likely transitions through development by mobilizing stored reserves through a suite of mitochondrial pathways independent of Complex I.

### Early onset of winter constrains energy gain in mountain pine beetle

Comparison of *D. ponderosae* larvae exposed to stepped cold during autumn (Fig. 4A,C,E) and winter (Fig. 4B,D,F) suggests that an early winter would be energy limiting and curtail an important period of energy acquisition. *Dendroctonus ponderosae* larvae exhibited distinct responses to the stepped-cold treatment in the autumn and winter, which suggests a shift from active metabolism in the autumn to energy conservation in winter. Protein content in larvae exposed to stepped cold in the autumn steadily decreased but returned to baseline after a 7 day recovery at −5°C (Fig. 4A). This transient protein loss may indicate increased protein turnover, protein use as an energy source or protein degradation to avoid the accumulation of dysfunctional protein that can occur in freeze-avoiding insects during early diapause (Hasanvand et al., 2020). In contrast, winter samples maintained stable protein levels during cold stress (Fig. 4B), suggesting an improved balance of protein turnover and stability in the dormant state. A noticeable decline in protein content in winter specimens during recovery at 6°C may point to protein mobilization for repair processes following cold stress (Colinet et al., 2009; Štětina et al., 2015).

Carbohydrate and lipid dynamics under cold stress further highlight seasonal shifts in metabolic strategy. In the autumn, a significant (∼60%) reduction in carbohydrate levels occurred as temperatures dropped from 6°C to 0°C and remained low throughout further cold exposure and recovery (Fig. 4C). A similar trend was observed in the winter time point (Fig. 4D), but the response appeared to shift at lower temperatures, and recovery at 6°C elicited a substantial rebound in carbohydrate content. The initial drops are consistent with findings from adult *D. ponderosae* (Thompson et al., 2020) as well as *Belgica antarctica* (Devlin et al., 2022), in which cold exposure triggered carbohydrate mobilization for cryoprotectant synthesis, a general strategy for freeze avoidance in insects (Zachariassen, 1985). The maintenance of consistently low carbohydrate content in *D. ponderosae* larvae in response to stepped cold stress in both the autumn and winter time points suggests that they initiate production of glycerol or other cryoprotectants early in response to cold stress. This mobilization likely helps in further suppression of the supercooling point, protection against macromolecular damage and enhanced metabolic flexibility, all of which contribute to cold tolerance (Marshall and Roe, 2021; Teets et al., 2023; Ullah et al., 2024).

In the autumn, lipids decline as the temperature decreases below 0°C (Fig. 4E), likely serving as a primary energy source to support ongoing metabolic demands in the absence of metabolic suppression at this time (Fig. S3C). Winter samples showed a slight increase in lipid content during cold exposure (Fig. 4F), suggesting a lipid-conserving strategy that would preserve long-term energy reserves for post-winter development (Enriquez and Visser, 2023; Han and Bauce, 1993).

Together, these findings underscore a key seasonal transition in *D. ponderosae*: from active metabolism and energy acquisition in the autumn to metabolic suppression and energy conservation in winter. In the autumn, exposure to cold appears to prompt the immediate use of carbohydrate reserves, likely to support basal maintenance and stress responses. By winter, however, *D. ponderosae* have shifted into a dormant and cold-tolerant state where extreme cold has less impact on reserve depletion, indicating metabolic suppression. These seasonally distinct responses highlight the critical role of metabolic suppression in their overwintering success and reveal pronounced physiological flexibility of *D. ponderosae* generally thought to be dormant and unresponsive at or below 6°C. Based on these results, we can say that *D. ponderosae* enter a state of winter dormancy under natural conditions; however, this dormancy appears to be regulated by a complex interplay of environmental and physiological cues, rather than temperature alone. Early onset of winter can limit their reserve gain and cause an energy strain due to lack of complete cold acclimatization, which possibly could affect their post-winter fitness. In contrast, mild winter temperatures might be beneficial for range expansion by providing greater time to feed and store energy for future use. Overall, *D. ponderosae* displays opportunistic behavior and strong physiological flexibility in coping with cold stress, adaptations which likely contribute to its success and potential for range expansion.

## Supplementary Material

10.1242/jexbio.251414_sup1 Supplementary information
