# Supplementary material for: Winter intensity shapes overwintering energy gain and use in bark beetles under range expansion
Source: J Exp Biol. 2026 Jan 30;229(2):jeb251414. doi: 10.1242/jeb.251414 (PMC12891943; doi:10.1242/jeb.251414)
Supplement: Supplementary information [file jexbio-229-251414-s1.pdf]

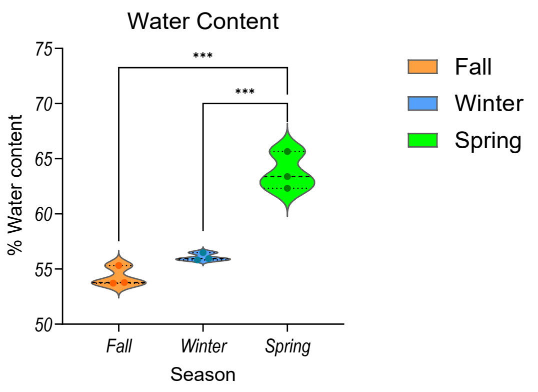

Fig. S1. Effect of seasonality on the water content of the mountain pine beetle using ANOVA. The water content was estimated in whole prepupae by drying them at 60°C for 7 days. The fall samples are presented in orange, winter samples are blue, and spring samples are green. Due to limited sample, 3 individuals were used per group. Statistical significance of p=0.001 are shown using ***.

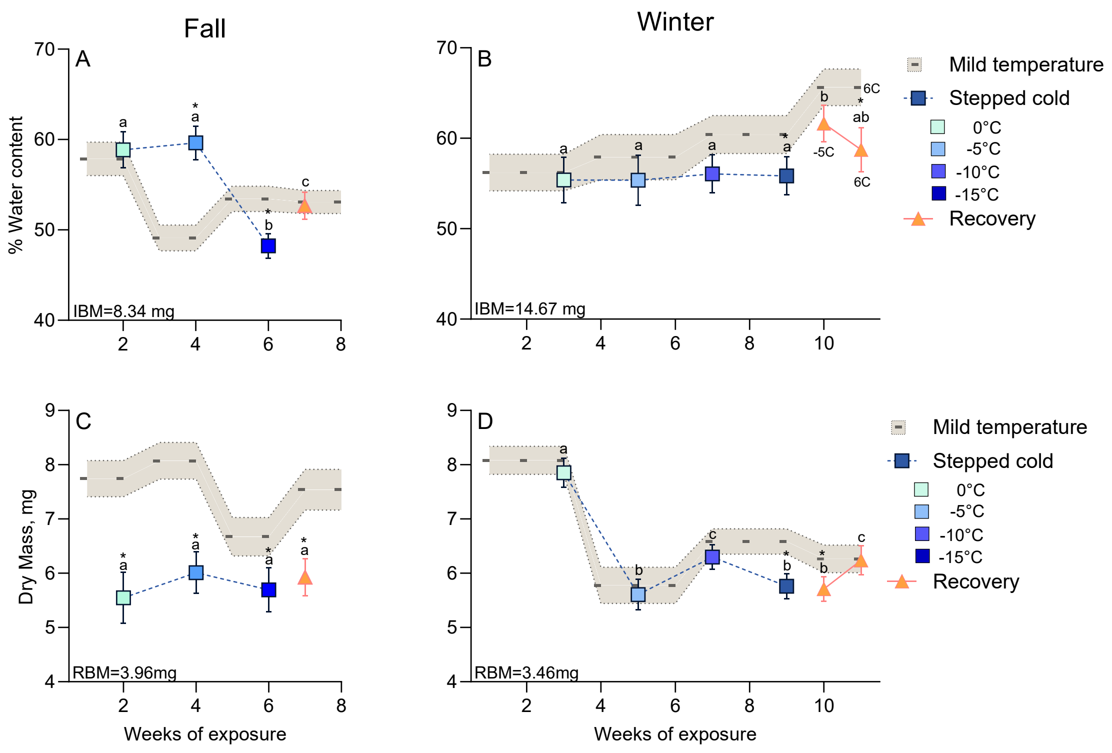

Fig. S2. Effect of mild temperature, stepped cold and recovery on the body composition of mountain pine beetle in the fall and winter seasons using linear model. The effect of mild temperature, stepped cold and recovery on the water content of mountain pine beetle in the fall and winter are shown in A & B, respectively. The initial body mass (IBM) was used as a covariate and is mentioned in the bottom left corner of the graphs. The effect of mild temperature, stepped cold and recovery on the dry mass of the mountain pine beetle in the fall and winter season is given in figures C & D, respectively. The residual dry body mass (RBM) for each macromolecule of interest was used as a covariate and is mentioned in the bottom left corner of each graph. The dashed line and beige band represent the mean and standard error (SEM), respectively, of macromolecule levels in mountain pine beetles maintained at a constant 6 °C (mild temperature group). The blue graduated color indicates the intensity of the cold stress as the temperature decreases from 0°C to -5°C, -10°C, and -15°C. The orange triangles show the recovery group at -5°C for both fall and winter and 7 days at 6°C in the winter group only. Recovery data for 7 days at 6°C was lost in the fall group due to technical errors. Each group in dry mass estimation contains 7-9 individuals and water content was estimated on 3 individuals per group. Different letters indicate the values differ significantly ($p<0.05$) within the cold-stressed group at different temperatures. Asterisks indicate the values differ significantly ($p<0.05$) from control group.

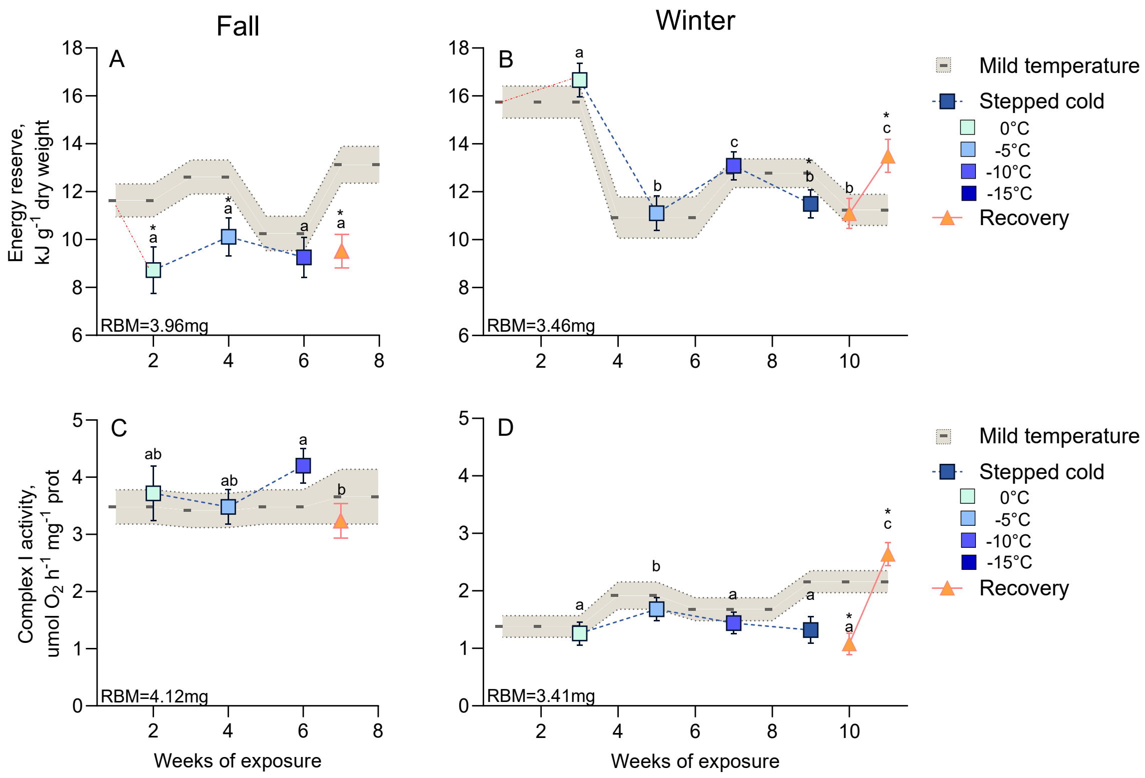

Fig. S3. Effect of mild conditions, stepped cold stress and recovery on the stored energy reserve and complex I activity of mountain pine beetle in the fall and winter seasons. The total tissue level energy reserve in samples from fall and winter season is shown in panel A & B, respectively. Panel C & D show the complex I activity of the electron transport system (ETS) of mountain pine beetle in the fall and winter seasons, respectively. The dashed line and beige band represent the mean and standard error (SEM), respectively, of macromolecule levels in mountain pine beetles maintained at a constant 6 °C (mild temperature group). The red dotted line shows the change when the temperature changed from 6°C to 0°C. The blue graduated color indicates the intensity of the cold stress as the temperature decreases from 0°C to -5°C, -10°C, and -15°C. The orange triangles show the recovery group at -5°C for both fall and winter and 7 days at 6°C in the winter group only. Recovery data for 7 days at 6°C was lost in the fall group due to equipment failure. The residual dry body mass (RBM) for each macromolecule of interest was used as a covariate and is mentioned in the bottom left corner of each graph. Different letters indicate the values differ significantly (*p*<0.05) within the cold-stressed group at different temperatures. Asterisks indicate the values differ significantly (*p*<0.05) from the control group.

Table S1. Data table for winter onset experiment: the macromolecules are presented as mg per individual, the energy reserve is presented as kJ g^{-1} residual dry mass, and complex I activity is presented as µmol O_2 h^{-1} mg^{-1} residual dry mass. Residual dry mass refers to dry mass excluding all macromolecules for energy reserve and complex I activity. The results are presented as mean ± SEM.

Temperature groups, °C	Protein		Carbohydrate		Lipid		Energy Reserve		Complex I activity	
	Fall	Winter	Fall	Winter	Fall	Winter	Fall	Winter	Fall	Winter
Mild temp, week 2	0.64 ± 0.06	0.88 ± 0.10	2.07 ± 0.21	1.86 ± 0.12	0.72 ± 0.07	1.58 ± 0.12	11.5 ± 0.71	16.1 ± 0.7	3.48 ± 0.3	1.38 ± 0.19
Mild temp, week 4	0.59 ± 0.06	0.83 ± 0.08	2.24 ± 0.22	1.01 ± 0.10	0.87 ± 0.07	0.69 ± 0.14	12.1 ± 0.71	11.2 ± 0.9	3.42 ± 0.3	1.92 ± 0.24
Mild temp, week 6(fall)/ 7 (winter)	0.79 ± 0.06	1.23 ± 0.08	1.34 ± 0.22	1.08 ± 0.11	0.76 ± 0.06	0.91 ± 0.1	10.4 ± 0.75	13.1 ± 0.6	3.48 ± 0.3	1.68 ± 0.2
Mild temp, week 8(fall)/ 10 (winter)	0.75 ± 0.06	1.21 ± 0.09	1.52 ± 0.22	1.18 ± 0.12	1.32 ± 0.07	0.54 ± 0.10	13.3 ± 0.79	11.5 ± 0.7	3.66 ± 0.3	2.16 ± 0.19
Stepped cold, 0°C	0.62 ± 0.08	0.89 ± 0.10	0.83 ± 0.31	1.32 ± 0.12	0.94 ± 0.09	1.94 ± 0.11	9.07 ± 0.99	16.9 ± 0.7	3.72 ± 0.48	1.26 ± 0.2
Stepped cold, -5°C	0.47 ± 0.06	0.94 ± 0.11	0.48 ± 0.25	0.32 ± 0.13	0.77 ± 0.06	1.13 ± 0.12	9.89 ± 0.81	12.4 ± 0.7	3.48 ± 0.3	1.68 ± 0.2
Stepped cold, -10°C	0.43 ± 0.06	1.13 ± 0.08	0.52 ± 0.23	0.34 ± 0.11	0.56 ± 0.07	1.13 ± 0.10	8.98 ± 0.86	13.4 ± 0.6	4.2 ± 0.3	1.44 ± 0.19
Stepped cold, -15°C	NA	1.08 ± 0.08	NA	0.26 ± 0.11	NA	0.82 ± 0.1	NA	11.7 ± 0.6	NA	1.32 ± 0.23
Recovery, -5°C	0.66 ± 0.05	1.18 ± 0.09	0.50 ± 0.22	0.23 ± 0.12	0.85 ± 0.06	0.74 ± 0.10	9.44 ± 0.72	11.0 ± 0.7	3.24 ± 0.3	1.08 ± 0.19
Recovery, 6°C	NA	0.98 ± 0.09	NA	0.88 ± 0.12	NA	1.18 ± 0.11	NA	13.8 ± 0.7	NA	2.64 ± 0.2

Journal of Experimental Biology • Supplementary information
